# Biophysical Aspects of Neurodegenerative and Neurodevelopmental Disorders Involving Endo-/Lysosomal CLC Cl^−^/H^+^ Antiporters

**DOI:** 10.3390/life13061317

**Published:** 2023-06-02

**Authors:** Maria Antonietta Coppola, Abraham Tettey-Matey, Paola Imbrici, Paola Gavazzo, Antonella Liantonio, Michael Pusch

**Affiliations:** 1 Istituto di Biofisica, Consiglio Nazionale delle Ricerche, 16149 Genova, Italy; maria.coppola@uniba.it (M.A.C.); masirlow@gmail.com (A.T.-M.); paola.gavazzo@ibf.cnr.it (P.G.); 2 Department of Pharmacy–Drug Sciences, University of Bari “Aldo Moro”, 70125 Bari, Italy; paola.imbrici@uniba.it (P.I.); antonella.liantonio@uniba.it (A.L.); 3 RAISE Ecosystem, 16149 Genova, Italy

**Keywords:** CLC proteins, endosome, lysosome, chloride transport, developmental

## Abstract

Endosomes and lysosomes are intracellular vesicular organelles with important roles in cell functions such as protein homeostasis, clearance of extracellular material, and autophagy. Endolysosomes are characterized by an acidic luminal pH that is critical for proper function. Five members of the gene family of voltage-gated ChLoride Channels (CLC proteins) are localized to endolysosomal membranes, carrying out anion/proton exchange activity and thereby regulating pH and chloride concentration. Mutations in these vesicular CLCs cause global developmental delay, intellectual disability, various psychiatric conditions, lysosomal storage diseases, and neurodegeneration, resulting in severe pathologies or even death. Currently, there is no cure for any of these diseases. Here, we review the various diseases in which these proteins are involved and discuss the peculiar biophysical properties of the WT transporter and how these properties are altered in specific neurodegenerative and neurodevelopmental disorders.

## 1. Introduction—The CLC Family

Physiologically, the most abundant anion is chloride. It is an important substrate of many transport proteins, being carried across the membrane as a single anion or coupled with other ions, and is important, for example, for the regulation of the membrane potential, intracellular vesicles acidification and cell volume regulation [1].

In humans, the CLC family is formed by nine members, which had initially been supposed to be all chloride channels, because of their sequence homology with the founding member, the Torpedo electroplax channel ClC-0 [2]. The discovery that the bacterial *Escherichia coli* ecClC-1 homologue is not a passive chloride channel but a stoichiometrically coupled secondary active 2 Cl^−^/1 H^+^ antiporter has dramatically changed the point of view of the entire CLC group [3]. Based on sequence homology, three branches of human CLCs have been distinguished. The first one includes the plasma membrane-localized chloride channels ClC-1, ClC-2 and the two isoforms ClC-Ka and ClC-Kb. The second branch is formed by ClC-3, ClC-4 and ClC-5, while the third branch contains ClC-6 and ClC-7. ClC-3 to -7 are all Cl^−^/H^+^ exchangers and are localized to the intracellular membranes of endosomes and/or lysosomes [1].

All CLC family members share the same dimeric architecture that is unique to this protein family. Except for ClC-6 and ClC-7 [4], the other CLC proteins can form homo- or hetero-dimers with members of the same branch [1]. Biochemical studies and single-channel analysis on the first cloned *Torpedo* ClC-0, mutants [2,5,6] and biochemical and low-resolution structural analysis of ecClC-1 [7,8] suggested a homodimeric “double-barreled” architecture, with physically separated anion transport pathways in each protomer. This architecture has been fully confirmed by the determination of ecClC-1 and *Salmonella typhimurium* stClC crystal structures [9,10]. The structures revealed the presence of distinct anion binding sites, formed by residues that are also highly conserved in human CLCs. The sites are denominated Sext, Scen and Sint, with Sext being occupied by the presumably negatively charged side chain of the “gating glutamate” E148 [9,10]. Each monomer presents 18 α-helices (from A to R) of which 17 are partially embedded in the membrane. The two subunits interact in a tight manner and the architecture follows an inverted and parallel orientation [1]. Two C-terminal tandem cystathionine-β-synthase (CBS) domains are present in most eukaryotic CLC proteins [11,12], but are absent in ecClC-1. The two CBS domains may have a role in the so-called common gating process (that will be discussed in more detail below) and confer unique features to the CLC members [1]. Dutzler and colleagues determined the crystal structures of isolated CBS domains of *Torpedo* ClC-0 [13], human ClC-5 [14] and human ClC-Ka [15]. CBS domains are present in many different protein families, where they are often implicated in the sensing of adenine nucleotides [11,16]. Structurally, so far, ATP has been found to be bound in the isolated domains of ClC-5 and in the full-length structure of ClC-7 [17], but not in isolated domains of ClC-0 and ClC-Ka and not in full-length structures of bovine ClC-K or human ClC-1 [18,19,20].

Single-channel recordings of the *Torpedo* ClC-0 channel displayed two kinds of gating mechanisms that regulate the open probability (P*o*) of the channel: a “*fast*” or “*protopore*” gate that acts independently on single pores determines the closing or opening state of each pore of the double-barreled structure [1]. The fast gate is mainly determined by the gating glutamate (E166 in ClC-0), in that its neutralization renders CLC-0 channels voltage independent. Protonation of the gating glutamate and its competition with permeant ions underlie the anion and pH dependent protopore gating of most CLC channels [10,21,22,23]. Conversely, a second mechanism, termed a *slow* or *common* gate, operates on both pores simultaneously and is still not well understood [1].

Some CLC proteins require association with a small ancillary subunit for proper function or membrane expression. In particular, the kidney ClC-Ka and ClC-Kb channels require association with the barttin subunit [24]. In glia cells, ClC-2 associates with GlialCAM, a protein with the typical architecture of a cell adhesion molecule, which is mutated in megalencephalic leukoencephalopathy with subcortical cysts (MLC) [25]. It leads to clustering of ClC-2 at glial cell–cell contacts and alters biophysical functions of the ClC-2 channel [25,26]. The complex of ClC-7 with its subunit Ostm1 is mandatory for mutual stabilization [4,27].

The plasma membrane localized chloride channels belonging to the first branch of the CLC family are expressed in a tissue-dependent manner that is different for each member according to their physiological role. All channel CLCs are involved in various human genetic diseases, as reviewed in detail elsewhere [1,28,29].

**Figure 1:**
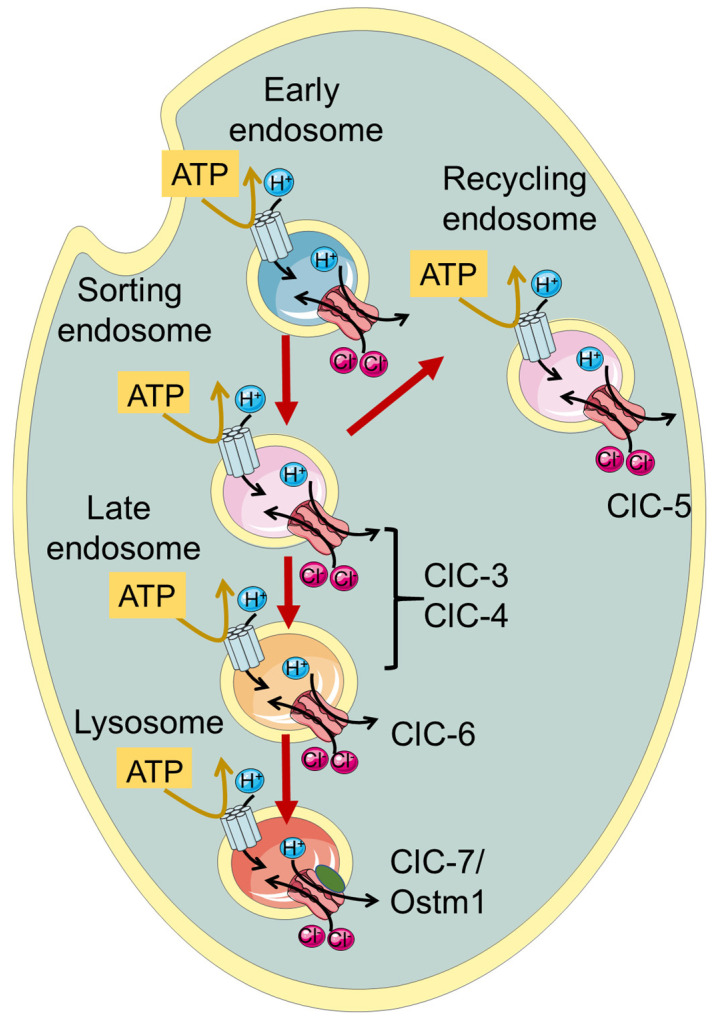
Schematic illustration of localization of vesicular CLCs in the endo-/lysosomal pathway.

ClC-3 through ClC-7, which are the focus of this review, function as Cl^−^/H^+^ exchangers and are localized to intracellular endosomes and/or lysosomes (Figure 1, Table 1). Initially, when the transporter function of the intracellular CLCs was not yet known, it was proposed that they act as charge-shunting chloride channels to assist the luminal acidification of endosomes and lysosomes intracellular organelles [30,31,32,33,34]. Indeed, the maintenance of an acidic pH of the lumen of endo-/lysosomes is required for their proper physiological function. The proton pumping V-ATPase is electrogenic and thus generates an electrical potential difference that would impede acidification if not neutralized by anionic cotransport and/or cationic counter-transport. Somewhat surprisingly and counter-intuitively, model calculations show that a 2 Cl^−^/H^+^ exchange activity, contributing to a more inside-negative voltage, allows a more acidic steady-state luminal pH compared to a shunting Cl^−^ channel [35,36].

Among the endo-/lysosomal CLCs, ClC-5 is rather specifically expressed in the kidney with a predominant presence in epithelial cells of the proximal tubule, where it is involved in endocytic uptake [30,31,33]. Indeed, mutations causing impaired ClC-5 transport activity are associated with Dent’s disease, a kidney disorder characterized by the primary symptom of low molecular weight proteinuria, and a series of secondary symptoms including kidney stones and renal failure, caused by defective endocytosis in the proximal tubule [33,37]. ClC-7, together with its subunit Ostm1 [27], is rather ubiquitously expressed in the body and is localized to lysosomes and in the ruffled border of osteoclasts functioning as a 2Cl^−^/H^+^ antiporter [1]. Accordingly, impaired bone resorption in osteoclast, caused by a functionally defective ClC-7/Ostm1 complex, causes osteopetrosis, a disease characterized by stiff and fragile bones [38].

**Table 1:** List of the CLC genes, subcellular localization and CLC related neurological diseases.

Protein	Gene	Cellular Localization	Neurological Disorder	Symptoms	References
ClC-3	*CLCN3*	Sorting and late endosomes	Global developmental delay	Intellectual disability, agenesis of the corpus callosum, epilepsy, visual impairment, hypotonia, anxiety, dysmorphic facial features	[39]
ClC-4	*CLCN4*	Sorting and late endosomes	*CLCN4*-related X linked intellectual disability syndrome	Intellectual disability, epilepsy, autism, growth and feeding difficulties, epilepsy, movement disorders, gastrointestinal conditions, dysmorphic facial features	[40,41,42]
ClC-6	*CLCN6*	Late endosomes			
			Early Onset Neurodegeneration	Severe neurodegeneration, severe generalized hypotonia and respiratory insufficiency, brain atrophy	[43]
			Kufs’ disease	Adult-onset neuronal ceroid Lipofuscinosis, movement and cognitive function impairment	[44,45]
			West syndrome	Severe developmental delay, autism, movement disorder, microcephaly, facial dysmorphism, visual impairment	[46]
ClC-7/ Ostm1	*CLCN7/* *OSTM1*	Lysosomes			
			Autosomal Recessive Osteopetrosis	Osteopetrosis, lysosomal storage disease, neurodegeneration, visual impairment	[38,47,48]
			Gain of function *CLCN7* related disease	Delayed myelination and development, organomegaly, and hypopigmentation	[49]

A large phenotypic spectrum of neuronal diseases is associated with mutations in the genes encoding ClC-3/-4/-6 and ClC-7, as will be described in detail in the following paragraphs (see Table 1).

For all vesicular CLCs, an unsolved question pertains to the direction of exchanger transport. Despite being physiologically localized to endo-/lysosomes, ClC-3 to -6 can reach the plasma membrane when heterologously expressed in HEK293 cells, allowing the investigation of their biophysical properties using the patch clamp technique [45,50,51,52,53]. For ClC-7, the elimination of N-terminal lysosomal targeting motifs leads to plasma membrane expression [4,54]. ClC-3 to -5 all exhibit extreme outward rectification of currents with very little or nonresolvable activation kinetics [50,51,52,53,55]. This current direction corresponds to the transport of luminal Cl^−^ out of lysosomes with a parallel influx of cytosolic H^+^. However, it remains unclear whether the direction of transport is physiologically relevant and whether CLC exchangers work synergistically with V-ATPase, contributing to luminal acidification.

Additionally, ClC-6 and ClC-7 exhibit strongly outwardly rectifying currents, which are, however, characterized by slow activation kinetics and measurable inward “tail” currents [4,45].

## 2. ClC-3 and ClC-4

The second branch of the CLC family comprises ClC-3, -4 and 5. These three endosomal transporters share high sequence similarity and have similar functional properties [1,29]. Among the human CLCs, they are the most similar to the *Escherichia coli* ecClC-1 homologue. The renal-specific ClC-5 is found mostly in recycling endosomes and its physiological role will not be discussed in detail [1]. ClC-3 and ClC-4 are localized to sorting endosomes, and ClC-3 is probably localized in late endosomes as well [1,29]. ClC-3 has also been proposed to play a role in synaptic vesicles. This is, however, still controversial and will not be discussed in detail here (see [29] for a discussion).

**Figure 2:**
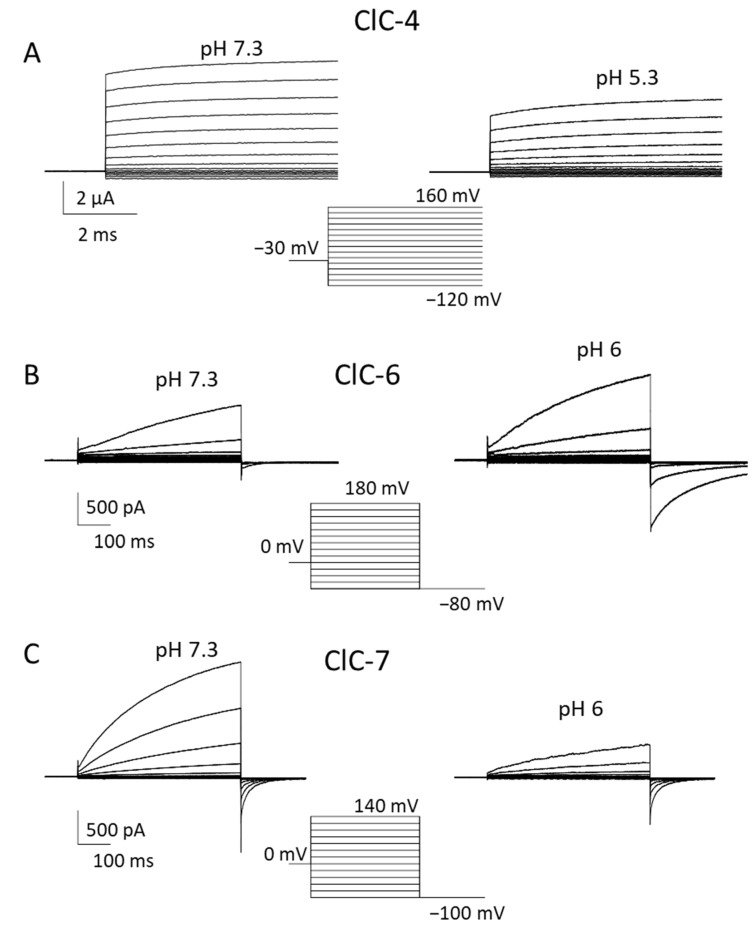
Biophysical properties of ClC-4, ClC-6 and ClC-7. Typical voltage-clamp current traces of ClC-4 ((**A**), expressed in *Xenopus* oocytes), ClC-6 ((**B**), expressed in HEK cells), and ClC-7^PM^ ((**C**), expressed in HEK cells), elicited by the indicated voltage-clamp protocols, measured using the two-electrode voltage clamp method (**A**) or the whole-cell recording mode of the patch clamp technique (**B**,**C**) at neutral (left panels) and acidic (right panels) pH. Extracellular solutions were 100 mM NaCl, 10 mM Hepes or MES, 5 mM MgSO_4_ (**A**) or 150 mM NaCl, 10 mM Hepes or MES, 4 mM MgSO_4_ (**B**,**C**). In B and C the pipette solution contained 130 mM NaCl, 10 mM Hepes, 2 mM EGTA, 2 mM MgSO_4_. ClC-3 is similar to ClC-4.

Functionally, these transporters are characterized by an extremely outwardly rectifying current–voltage relationship with almost instantaneous activation at positive voltages [50,51,52,55,56,57,58] (Figure 2A). The extreme outward rectification precluded the determination of transport stoichiometry by reversal potentials measurements [59]. Using a fluorescent assay, a 2 Cl^−^/1 H^+^ stoichiometry was determined for ClC-5, which is probably similar for ClC-3 and ClC-4 [55]. The outward rectification at least partially reflects a gating process, as evidenced by a single point mutation (D76H) that conferred detectable inward tail currents to ClC-5 [60]. The mutant also allowed us to measure reversal potentials for ClC-5 for the first time, confirming the 2:1 Cl^−^/H^+^ transport stoichiometry [60].

Most evidence on the physiological roles of ClC-3 and ClC-4 was obtained from mouse models and their involvement in human genetic diseases. ClC-3 KO mice display severe postnatal neurodegeneration with almost total loss of the hippocampus after 3 months [34,61,62]. Yet, ClC-3 KO mice have a normal life span. Neurodegeneration is unlikely to be caused by impaired synaptic function [1,29]. Another set of mouse models showed that ClC-4 protein stability relies on the presence of ClC-3 via heterodimerization [63,64]. While ClC-4 KO mice have no overt phenotype, the double KO of ClC-3 and ClC-4 has a more severe phenotype than CLC-3 KO mice [64]. Notably, differently from humans, *Clcn4* is not X-linked in laboratory mice. For this reason, a rat model might be more useful for the investigation of mechanisms underlying *CLCN4*-related disease, because in rats, as in humans, *Clcn4* is X-linked.

While ClC-4 KO mice have no overt phenotype, in 2013 and 2016, patients (mostly pediatric) with a range of neurodevelopmental and psychiatric complications have been described with X-linked *CLCN4* variants [40,42,65] (see Table 1). In heterologous expression, these and some novel variants [66] showed variable loss of function effects. It is important to note that complete loss of ClC-4 protein leads to non-syndromic intellectual disability in males and no disease in heterozygous females. In contrast, de novo and inherited missense variants can lead to severe syndromic neurological disease in males as well as in females, suggesting a dominant effect. In a recent study, a large number of *CLCN4* families was investigated, describing a large spectrum of clinical phenotypes and studying > 50 missense variants in heterologous expression [41]. Novel biophysical mechanisms were discovered for new and already described variants. These included a toxic gain of function characterized by the presence of negative currents at acidic extracellular (luminal) pH, and a shift in the voltage dependence of gating to more positive voltages [41]. Both effects can be expected to exert dominant negative effects in ClC-3/ClC-4 heterodimers.

Almost simultaneously came the discovery of the first variants in *CLCN3* that cause global developmental delay, intellectual disability and neurodevelopmental disorders [39] (Table 1). Detailed functional analysis revealed a toxic gain of function for two missense variants, similar to the above-described effects in some *CLCN4* variants [39].

## 3. ClC-6

The third branch of the CLC family comprises ClC-6 and ClC-7, which both function as Cl^−^/H^+^ exchangers [1,45]. Even though the expression of ClC-6 mRNA appears to be ubiquitous in many tissues [67], biochemical analysis detected native ClC-6 protein predominantly in neurons, where it localizes to late endosomes and partially lysosomes [44]. For a long time, the biophysical profile of ClC-6 remained completely unknown. In the first attempts at heterologous expression, no currents attributable to ClC-6 could be detected [67], possibly caused by the intracellular localization of most of the overexpressed protein [68].

A subtype of lysosomal storage disease, referred to as neuronal ceroid lipofuscinosis (NCL), was observed in ClC-6 *knockout* mice presenting a mild phenotype with features of reduced pain sensitivity, probably due to strong accumulation of materials in axon initial segments, mild cognitive abnormalities and no impact on their span life [44]. This evidence suggested that *CLCN6* variants could be involved in human NCL [44]. Indeed, in a sample of 75 adult-onset variants, including late-onset forms of NLC and Kufs’ disease, two individuals were found to be heterozygous for *CLCN6* missense variants (V580M and T628R) [44]. However, no functional analysis had been performed at the time of that study because the transporter had not been successfully functionally characterized.

Preliminary electrophysiological characterization was obtained when the N-terminus of ClC-6 tagged with GFP (GFP-ClC-6) was reported to enhance its cell surface localization [69]. However, the reported currents were small and barely above background levels.

In 2020, the same de novo variant in *CLCN6* leading to the amino acid change Y553C was reported in three pediatric patients with no parental correlation [43]. The patients exhibited a severe syndrome characterized by early-onset neurodegeneration. The mutated tyrosine, located in the extracellular P-Q loop, is highly conserved among CLC antiporters. Electrophysiological measurements of ClC-6^Y553C^ revealed large currents activated at positive voltages (≥60 mV), representing a clear gain of function effect [43]. HEK cells overexpressing the mutant showed a dramatic vacuolization [43]. The luminal pH of these organelles did not reach low values, suggesting that the mutant impairs endolysosomal ionic homeostasis in patients.

In 2022, Zifarelli et al. were able to obtain robust recordings of WT ClC-6 currents by applying very large positive voltages beyond 140 mV [45,70]. Interestingly, the magnitude and properties of currents were independent of the N-terminal GFP tag. Similar to ClC-7, ClC-6-mediated currents showed slow activation at positive voltages; however, they required voltages of at least 140 mV to measure appreciable amplitudes [45] (Figure 2B). The necessity of the large voltages explained why these currents had escaped detection so far. Currents represent the coupled Cl^−^/H^+^ antiport, as assayed by reversal potential measurements [45]. Similar to ClC-7 [71], ClC-6 also exhibits so called “transient” or “capacitive” currents, whose possible physiological role is, however, obscure [45]. Interestingly, neutralizing the so-called proton glutamate did not completely abolish transport currents [45]. In contrast to most other CLC proteins, ClC-6-mediated currents were enhanced at acidic pH (6.3) compared to neutral pH [45] (Figure 2B), a finding that is of likely physiological relevance. In light of these novel findings on the functional properties of ClC-6, Zifarelli et al. performed a reexamination of the disease-causing variant ClC-6^Y553C^, concluding that the mutation causes a gain of function by “shifting” the voltage dependence of ClC-6 gating to less positive voltages [45]. Possibly, Y553, being localized at the subunit interface of the homodimer, is involved in the common gating mechanism that acts on both ion-transporting units of the dimer.

The discovery of suitable recording protocols allowed Zifarelli et al. to study the functional impact of the two above-mentioned variants found in Kufs’ disease patients. While T628R was indistinguishable from WT ClC-6, precluding firm conclusions regarding its causative nature for the disease, variant V580M showed a clearly reduced function, suggesting a causal relationship [45]. Since the variant was found in heterozygosity, it might exert a dominant negative effect in WT/mutant heterodimers.

Patients with the completely unrelated West syndrome, characterized by epilepsy, among other symptoms, have been reported to carry the ClC-6 E200A variant [46]. E200 is the critical gating glutamate of the exchanger and it is known that its neutralization in all studied vCLCs, including ClC-6, eliminates H^+^ transport and transforms it into an uncoupled ohmic chloride channel [69]. In a heterologous expression system, ClC-6^E200A^ caused an impairment of the autophagosome-mediated degradation system, likely because the fusion with lysosomes was compromised [46].

The three classes of disease related *CLCN6* mutations, i.e., gain of function (Y553C), reduction of function (V580M), and uncoupling (E200A), have different effects on the functional properties of the ClC-6 antiporter, leading to clinical phenotypes with different degrees of severity. In particular, ClC-6^Y553C^ causing a gain of function is associated with drastic neurodegeneration, whereas ClC-6^E200A^ could be defined as a loss of function in the respect of the uncoupling transport generated and related to a mild phenotype.

The recent remarkable progress that has been made regarding the functional analysis of ClC-6 activity will allow us to decode further mechanisms underlying disease caused by defective ClC-6 proteins.

## 4. ClC-7

Belonging to the third mammalian CLC branch, ClC-7 shares 45% of sequence homology with ClC-6. It was cloned in parallel with ClC-6 in 1995 [67], but could not be functionally analyzed for a long time. Intriguingly, ClC-7 is the only subcellular CLC member to be present almost exclusively in lysosomes [44]. Moreover, it has also been found in the ruffled border of osteoclasts, where it participates in bone resorption [38]. Unlike the other CLC transporters, ClC-7 requires association with a type I transmembrane protein, called Ostm1, for proper function and stability [4,27].

Similarly to ClC-6, no information about electrophysiological ClC-7 characterization has been available for a long time, due to its intracellular localization upon heterologous expression [1]. Ion flux studies with isolated mouse lysosomes showed that ClC-7 is the dominant anion permeation pathway of lysosomal membranes and that it performs 2 Cl^−^/1 H^+^ antiport activity [72]. A breakthrough was achieved by Stauber and Jentsch, who discovered the sorting motifs that mediate lysosomal targeting [54]. In particular, they found that when four leucine residues localized in the N-terminal portion are changed to alanine, the transporter is at least partially targeted to the plasma membrane [54]. Notably, Ostm1 follows ClC-7 in its expression location. ClC-7^PM^, the ClC-7 variant in which the two dileucine motifs are mutated to alanine, elicited robust transmembrane, outwardly rectifying voltage-activated currents [4] (Figure 2C). Even though some electrophysiological properties of ClC-7 are similar to that of other vesicular CLCs, including the inhibitory effect of acidic pH, ClC-7 differs substantially from ClC-3 to -5. Most importantly, ClC-7^PM^ exhibits very slow activation kinetics in the seconds time range [4] (Figure 2C). This slow “gating” phenomenon is strictly linked to conformational changes in the proteins, where the interactions between transmembrane as well as cytoplasmic domains play a key role [73]. In addition to the transport currents, Pusch and Zifarelli discovered that the transporter also exhibits rather large “transient” or “capacitive” currents that reflect charge rearrangements within the protein. These are most likely mediated by movements of the gating glutamate and chloride binding/unbinding events [71]. Similar currents have been observed in ClC-5 and ClC-3 [52,74,75]. The transient currents probably have no physiological role, but represent a biophysical feature that can be useful in deciphering molecular mechanisms of gating and transport. Interestingly, while in ClC-5, neutralization of the so-called proton glutamate completely abolished transport currents, leaving only transient currents [74,75], in ClC-7, residual transport currents were observed in the corresponding E312A mutant [71].

The physiological role of ClC-7 remained unclear for a long time. The first insights were obtained with a mouse KO model that was characterized by severe osteopetrosis [38]. The involvement of ClC-7 in bone resorption was confirmed by the presence of *CLCN7* mutations in a human patient with malignant osteopetrosis [38]. Further evidence came from the identification of a spontaneous Ostm1 mutation to be associated with the onset of a severe osteopetrosis in *gray lethal* mice presenting a fur color defect [76]. In Clcn7^−/−^ mice, even though the number of osteoclasts was normal, their ability to reabsorb calcified bone was impaired [38]. Interestingly, however, no impact on lysosomal acidification was observed, suggesting that the osteoclasts’ ability to acidify intracellular vesicles was preserved in Clcn7^−/−^ mice [38]. The life span of the KO mice was limited to 6–7 weeks.

Importantly, in addition to osteopetrosis, ClC-7^−/−^ mice also presented severe lysosomal storage associated with central nervous system and retinal degeneration [47]. Using a lacZ fusion protein, the expression profile of ClC-7 was determined in the nervous tissue of WT e KO mice revealing the hippocampus CA3 region, the cortex and the cerebellum as the main regions experiencing neuronal loss in KO mice [47]. Electron microscopy analysis revealed the presence of autofluorescent lipopigment in the regions affected by neurodegeneration and deficient of CLC-7 [47]. This factor, together with the detection of microglial activation and astrogliosis, represents three important hallmarks of neuronal ceroid lipofuscinosis (NCL) [47]. Importantly, no significant difference in pH values in lysosomes of cultured neurons and fibroblasts was found in Clnc7^−/−^ mice, but rather a reduction in lysosomal Cl concentration was observed [47]. Several pieces of evidence suggest that osteopetrosis and neurodegeneration are independent outcomes. First, an osteopetrotic mouse model with a mutation in the a3 subunit of V-type H^+^-ATPase (oc/oc mice) does not show retinal or neurodegeneration [47]. Moreover, the osteoporotic phenotype in Clnc7^−/−^ mice could be rescued by transgenically expressing ClC-7 in osteoclasts and macrophages under the control of tartrate-resistant acid phosphatase (TRAP) promoter [47]. This treatment achieved a lifespan increase, but it was not enough to ensure their survival due to the enduring neurological problems [47]. Surprisingly, the same approach failed when TRAP promoter-mediated Ostm1 expression was applied to rescue osteopetrosis in gray lethal (gl) mice which also displayed neuronal loss [77]. The severity of the phenotype and the short life span of the mice were serious problems, preventing a better understanding of the mechanisms underlying the progression of neurodegeneration in lysosomal pathologies [1]. The first information was collected when Wartosch et al. designed a floxed *Clcn7* mouse model allowing tissue-specific ClC-7 depletion [78]. No difference in lifespan between neuron-specific Clcn7 KO and WT mice was observed. Moreover, neuron-specific Clcn7 KO mice had no osteopetrotic phenotype; thus, the quality of life of these mice was improved compared to Clcn7^−/−^ [78]. Importantly, it was observed that neuronal loss occurs in regions, where ClC-7 had been disrupted and neurodegeneration started in the CA3 region of the hippocampus as in constitutive Clcn7^−/−^. Accordingly, in previous studies, astrogliosis and microglia activation were observed in the regions lacking ClC-7. Impaired lysosomal protein degradation was suggested after the detection of increased levels of LC3-II, a marker of autophagy [78].

A somewhat surprising finding was that several, mostly dominantly inherited, *CLCN7* variants causing osteopetrosis (but not neurodegeneration) produce a significant acceleration of gating kinetics [4,79,80]. It is unclear how this biophysical defect is related to ClC-7 malfunction.

More recently, a completely different disease characterized by delayed myelination and development, organomegaly and hypopigmentation was found in two children who both carried the de novo Y715C variant [49]. Surprisingly, none of the patients showed osteopetrosis (see Table 1). The variant, located in the C-terminus, was associated with larger currents when directed to the plasma membrane, representing a clear gain of function effect. Interestingly, the lysosomes of patient fibroblasts were enlarged and had a lower pH (0.2 pH units) than control lysosomes [49]. Even more excitingly, in the ClC-7 structure, Y715 is relatively close to the bound PI(3)P molecule (see below) and Leray et al. recently reported that intracellular phosphatidylinositol-3,5-bisphosphate (PI(3,5)P2) appears to tonically inhibit ClC-7 function, and that the Y715C variant was insensitive to PI(3,5)P2 [81]. The regulation of CLC-transporters by these signaling molecules is clearly an exciting aspect that needs to be understood in more detail.

The structure of the ClC-7/Ostm1 complex has been determined by two independent groups [17,82]. The structures revealed that the heavily N-glycosylated luminal region of Ostm1 forms a sort of cap on the luminal portion of ClC-7, preventing its degradation by lysosomal proteases [17,82]. Importantly, the mutual protein stabilization between ClC-7 and Ostm1 is suggested by the observation that the gray lethal mouse line, which lacks Ostm1, showed very weak ClC-7 staining; similarly, in Clcn7^−/−^ mice, there was only weak Ostm1 staining [27,76]. Both cryo-EM structures revealed strong intramolecular interactions between the cytosolic N-terminal portion and the CBS domains [17,82]. This important feature is also conserved in ClC-6 (Hite, personal communication), but the role of these interactions in other CLCs remains unclear because no information about the cytosolic structure of other CLC members is available. Similarly to ClC-5, the ClC-7 CBS domains bind ATP and additionally, a Mg^2+^ ion was found to be bound [17,82]. However, ATP had no effect on transport activity and its role remains to be understood [4]. Moreover, in the structure of ClC-7 an endolysosomal phosphatidylinositol 3-phosphate (PI3P) lipid was found to be bound at the interface between the CBS and the membrane domains [17].

## 5. Conclusions

While significant progress has been made in elucidating the functional properties of vesicular CLC transporters and their involvement in various neurological diseases, highlighting their importance in nervous system development and homeostasis, their precise physiological role is still largely unknown. Even for the most studied ClC-7, it is still disputed whether it is primarily necessary for proper luminal acidification or the regulation of the luminal chloride concentration. Most recent evidence favors the idea that ClC-7 is responsible for achieving a high luminal chloride concentration, which is important for phagosomal clearance [83]. Less clear are the roles of ClC-3 and ClC-4 in endosomes and the possible involvement of ClC-3/ClC-4 dimers in human genetic diseases. For all vesicular CLCs, and in particular for ClC-6, the significance of the activation at highly positive voltages remains enigmatic, since similar voltages are not expected to be achieved in endosomes. However, it is clear that ClC-3 and ClC-4 need to be inactive at negative voltages, since even a small amount of activity at these voltages, caused by gate disrupting mutations, leads to severe disease for both transporters [39,41]. In general, it appears that gain of function mutations lead to more severe phenotypes than loss-of function mutations for all vesicular CLCs [39,41,43,49]. In this respect, specific pharmacological inhibitors of vesicular CLCs are highly desirable. Unfortunately, thus far, no useful pharmacological tools are available for any of them.

## Data Availability

Data of Figure 2 are available upon reasonable request.
